# Tunable Electrical Conductivity of Carbon-Black-Filled Ternary Polymer Blends by Constructing a Hierarchical Structure

**DOI:** 10.3390/polym9090404

**Published:** 2017-08-31

**Authors:** Qiyan Zhang, Bo-Yuan Zhang, Zhao-Xia Guo, Jian Yu

**Affiliations:** Key Laboratory of Advanced Materials (MOE), Department of Chemical Engineering, Tsinghua University, Beijing 100084, China; qiyanzh@163.com (Q.Z.); boyuanzhang@126.com (B.-Y.Z.)

**Keywords:** polymer composites, hierarchical structure, electrical conductivity, ternary blends, carbon black

## Abstract

A type of hierarchical structured composite composed of a minor thermoplastic polyurethane (TPU) phase spreading at the interface of two major phases polyoxymethylene/polyamide copolymer (POM/COPA) and carbon black (CB) particles selectively localized at the TPU/COPA interface of the tri-continuous blends was fabricated by melt compounding. The hierarchical structure was designed according to predictions and verified by a combination of electron microscopy and solvent extraction technique. The hierarchical structured composites show the dramatically decreased percolation threshold, a reduction of 60% compared to those without TPU where CB is selectively distributed in the COPA phase. The effects of CB contents and TPU on the phase morphology of POM/COPA were investigated, showing the occurrence of the POM/COPA phase inversion from a sea-island to a co-continuous structure beyond the percolation threshold of CB in the presence of TPU. The mechanism for the formation of conductive network is construction of CB network at the TPU/COPA interface of tri-continuous POM/COPA/TPU blends and double percolation effect.

## 1. Introduction

Polymer blends have been an important research subject for decades because of their ability to combine the unique properties of individual components and the low processing cost compared to the development of new polymers [1]. Multi-phase (more than two phases) immiscible polymer blends are of high interest currently [2,3,4,5,6,7,8,9,10,11,12,13,14]. In addition to developing new high-performance materials, one more important reason is recycling of waste plastics in dealing with the environmental concern because it is hard to separate different types of plastics from the waste stream.

Incorporating functional fillers into ternary polymer blends can produce materials with added values and widen their application fields. CB is a widely commercialized conductive filler and also a good colorant. It is an ideal candidate to be incorporated into waste plastic mixtures during the recycling process, producing black antistatic or conductive materials. Furthermore, rational use of the multiphase morphology of ternary blends to fabricate CB-based conductive polymer composites can dramatically reduce the percolation threshold of CB compared to the relatively simple structure of the corresponding binary blends. In fact, some work has been reported [15,16,17,18,19,20,21,22,23,24,25]. For example, Al-Saleh and Sundararaj [15,16,17] investigated CB-filled polypropylene/polystyrene (PP/PS, 70/30)/styrene-butadiene-styrene (SBS, 5 vol %) ternary blend and found that the electrical percolation threshold reduces by 40% compared to that of the corresponding CB-filled PP/PS (70/30) binary blends. Shen et al. [20] investigated CB-filled poly(methyl methacrylate) (PMMA)/PP/ethylene-acrylic acid copolymer (EAA) ternary blends and found that their electrical percolation threshold is only one-fifth that of PP/CB composites. So far, the reported work only involves a few blend systems. All the work deals with tri-continuous hierarchical structures composed of two major phases and a copolymer that has certain compatibility with the two major components as minor phase spreading at the interface of two major phases, and CB particles selectively localized in the minor component. Strictly speaking, the conductive filler is not localized at the interface of the two major phases, it is just localized in the interphase. Although such structure shows high efficiency [15,16,17,18,19,20,21,22,23,24,25,26,27,28], if the conductive filler could be localized at the interface of the minor phase and one major phase in the ternary blends, the percolation threshold would reduce more. If ordinary polymers could be used as the minor component, the types of blend systems could be widened.

Recently, our research group found that CB particles can stably localize at the interface of TPU/COPA blends because the CB particles hydrogen-bonded with both TPU and COPA can act like Janus particle-type compatibilizers [26]. In this work, CB-filled POM/COPA/TPU ternary polymer blends are investigated to prepare POM-based conductive composites at low CB content through structure design. A hierarchical structure composed of minor TPU component spreading at the interface of two major components (POM/COPA) and CB particles selectively localized at the COPA/TPU interface is confirmed by electron microscopy. The reduction of the percolation threshold, the phase morphology evolution as a function of CB content, and the importance of TPU in phase morphology evolution are investigated to show the improvement in electrical conductivity and the mechanism for the improvement.

## 2. Materials and Methods

### 2.1. Materials

All the materials used in this work are commercially available. POM (M90), with a density of 1.41 g·cm^−3^, was provided by Yuntianhua Group Co. (Yunnan, China). It is a copolymer type with a melt flow index of 9.0 g/10 min. COPA (NT170) was obtained from Sailulu Industries, Ltd. (Shanghai, China). TPU (WHT1180, polyester type) with a density of 1.18 g·cm^−3^ was purchased from Wanhua Chemical Group Co. (Shandong, China). CB (VXC500), with a dibutyl phthalate (DBP) absorption of 1.48 cm^3^/g, and an iodine absorption of 74 mg/g, was purchased from Shanghai Cabot Chemical Co., Ltd. (Shanghai, China). Its particle size is about 25 nm, measured by TEM. The TPU and COPA pellets were dried in a vacuum oven for 12 h at 80 and 110 °C, respectively, before use.

### 2.2. Preparation of the Composites

Composites were prepared by melt mixing in a torque rheometer (RM-200A Rheometer, Harbin Hapro Electrical Technology Co., Harbin, China) at 60 rpm and 200 °C for 5 min. The melt mixed samples were cut and compressed into disk in a hot press (Labtech Engineering Co., Ltd., Samutprakarn, Thailand) at 50 bar and 200 °C for 5 min for different testing. In this work, two sets of samples were prepared first: POM/COPA/CB (70/30/*x*) and POM/COPA/TPU/CB (65/30/5/*x*), where *x* is CB content expressed in parts per hundred resin (phr). Then, the composition ratio of POM and COPA was varied keeping the CB and TPU contents constant at 6 and 5 wt %, respectively. For contrast, the samples without TPU were also prepared at constant CB loading of 6 wt %. The samples were denoted as POM/6CB/*y*COPA and POM/6CB/*y*COPA/5TPU, where *y* represents the weight fraction of COPA.

### 2.3. Characterization

The surface tension and its polar and disperse components of COPA were deduced by measuring the contact angle between the surface of COPA film and two testing liquids: deionized water and methylene iodide. Contact angles were measured using a HARKE-SPCAX3 model apparatus (Beijing Ha Ke Test Instrument Factory, Beijing, China). The surface tension at ambient temperature (20 °C) was calculated according to the Owens-Wendt geometric mean equation [29,30]. The data at the processing temperature were extrapolated from the ambient temperature data using a temperature coefficient [29].

A ZC-36 Resistivity Test (Shanghai 6th electrical meter factory, Shanghai, China) was used to measure the samples (diameter 75 mm and thickness 0.375 mm) with high resistivity (higher than 10^8^ Ω·cm). Samples (diameter of 30 mm and thickness of 2.5 mm) were tested using an ACL 800 Megohmmeter (ACL Incorporated, Chicago, IL, USA) for resistivity between 10^4^ to 10^8^ Ω·cm and a KDY-1 Resistivity Test (Guangzhou Kunde Technology Co., Guangzhou, China) based on the four-point method for resistivity lower than 10^4^ Ω·cm. All the resistivities reported in this paper are volume resistivities, and data are averages of four measurements.

The morphologies of the samples and the distribution of CB particles in the composites were examined by using a FESEM (JSM-7401, JEOL Instrument, Tokyo, Japan) and a TEM (H-7650, Hitachi Instruments, Tokyo, Japan). FESEM samples were cryo-fractured in liquid nitrogen, and some of them were immersed in *N*,*N*-dimethyl formamide (DMF) or m-cresol for 4 h to etch the TPU phase or COPA phase, and dried thoroughly before FESEM observation. The accelerating voltage of FESEM was set at 3 kV. The operating voltage of TEM was set at 200 kV and samples were cut into ultrathin films using an ultratome before observation.

## 3. Results and Discussion

### 3.1. Predictions of Blend Morphology and CB Localization

In addition to the formation of the tri-continuous structure that is composed of minor phase spreading at the interface of the two major phases, there are three other morphologies, i.e., the minor component is dispersed as droplets inside one of the two major phases or localized at the interface of the two major phases as partial wetting, that are also possible in this kind of ternary polymer blend [10,11]. In general, the morphology of a ternary polymer blend can be predicted by using a set of three spreading coefficients, each giving the tendency of one component to spread at the interface of the other two [11]:

(1) $$\lambda_{\mathrm{ikj}}=\gamma_{\mathrm{ij}}-\gamma_{\mathrm{ik}}-\gamma_{\mathrm{kj}}$$

where λ_ikj_ is the spreading coefficient indicating the tendency of component k to spontaneously spread at the interface of component i and component j, and it would be happen when the λ_ikj_ is positive. γ_ij_, γ_ik_, and γ_kj_ are the interfacial tensions for each polymer pairs. The interfacial tension can be obtained by using the harmonic mean equation [29]:

(2) $$\gamma_{\mathrm{ij}}=\gamma_{i}+\gamma_{j}-\frac{4\gamma_{i}^{d}\gamma_{j}^{d}}{\gamma_{i}^{d}+\gamma_{j}^{d}}-\frac{4\gamma_{i}^{p}\gamma_{j}^{p}}{\gamma_{i}^{p}+\gamma_{j}^{p}}$$

where γ_i_ and γ_j_ are the surface tensions of components i and j, γ_i_^d^ and γ_j_^d^ are the dispersive parts of the surface tensions of components i and j, γ_i_^p^ and γ_j_^p^ are the polar parts of surface tensions of components i and j. On the basis of the surface tension values (Table 1), the interfacial tensions for the pairs in this study were calculated by Equation (2) and the results are listed in Table 2.

For the POM/COPA/TPU ternary blend to take the desired morphology, where the minor TPU phase is spread at the interface of the two major phases (POM, COPA), the spreading coefficient for the TPU needs to be positive, and the spreading coefficients of the POM and COPA need to be negative. The spreading coefficients of each component listed in Table 2 and calculated by Equation (2) shows that λ_POM/TPU/COPA_ > 0, λ_POM/COPA/TPU_ < 0, λ_TPU/ POM/COPA_ < 0, which indicated that the minor TPU phase spreading at the interface between the POM and COPA to form a tri-continuous structure is possible from a thermodynamic point of view.

On the other hand, the selective distribution of fillers in an immiscible polymer blend can be predicted using the wetting coefficient, when the thermodynamic equilibrium is achieved in the system. The wetting coefficient of CB (ω_ij_ ) in a binary polymer blend can be calculated using Young’s equation [34]:

(3) $$\omega_{\mathrm{ij}}=\frac{\gamma_{\mathrm{CB}-i}-\gamma_{\mathrm{CB}-j}}{\gamma_{\mathrm{ij}}}$$

Where γ_CB-i_ and γ_CB-j_ are the interfacial tensions between CB and the two polymers component i and j. When ω_ij_ > 1, CB will be preferentially localized in the j phase. When –1 < ω_ij_ < 1, CB tends to localize at the interface of the blend. When ω_ij_ < −1, CB will be preferentially localized in the i phase.

The results of the calculated wetting coefficients and CB localizations are summarized in Table 2. It indicates that CB tends to selectively localize in the COPA phase of POM/COPA blend, in the TPU phase of POM/TPU blend, and in the COPA phase of TPU/COPA blend. The actual localizations of CB in POM/TPU and TPU/COPA have been reported [26,35]. The former is in agreement with the prediction from the wetting coefficient, while the latter is different because of another thermodynamic reason. CB particles can adsorb both TPU and COPA molecules through hydrogen bonding and, thus, localize at the TPU/COPA interface, acting like Janus particle-type compatibilizer [26].

Based on the above discussion, composites composed of a ternary POM/COPA/TPU blend as the matrix and CB as the filler have the potential to form a hierarchical structure, where minor TPU component spreading occurs at the interface of two major components POM and COPA and CB particles are selectively localized at the interface of COPA and TPU.

### 3.2. Experimental Evidence for Blend Morphology and CB Localization

**Figure 1:**
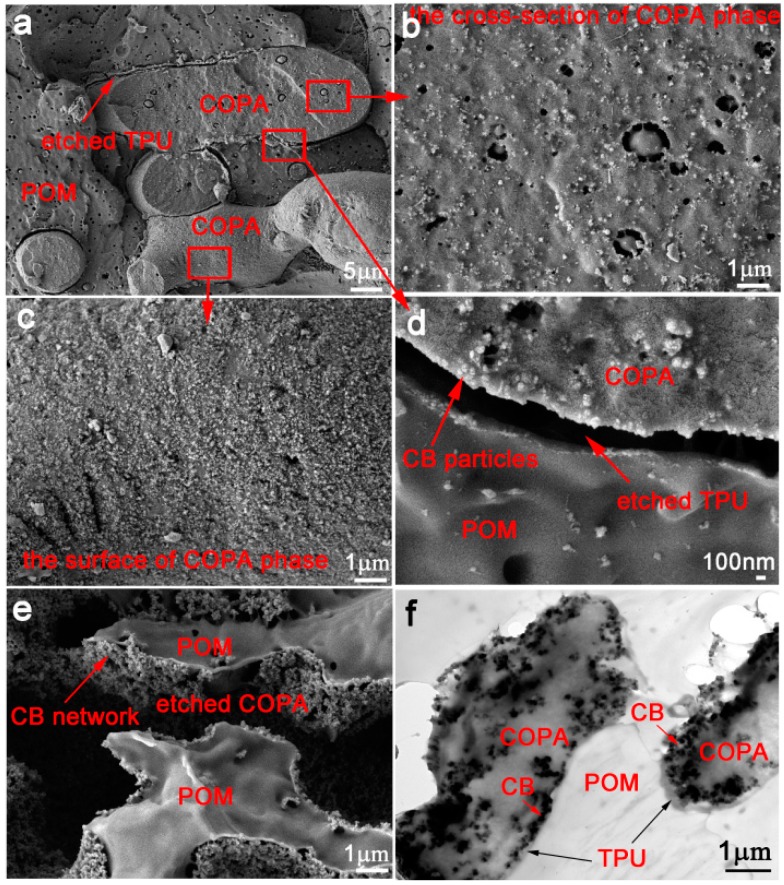
FESEM (**a**–**e**) and TEM (**f**) images of POM/COPA/TPU/CB (65/30/5/6), in which TPU phase were etched by DMF (**a**–**d**) and COPA phase are etched by m-cresol (**e**).

FESEM and TEM images for the sample POM/COPA/TPU/CB (65/30/5/6) are shown in Figure 1 to provide evidence for phase morphology of the ternary blend and localization of CB particles. Figure 1a shows the FESEM image of the cryofractured cross-section of the sample at relatively low magnification, where the TPU phase was etched by DMF for increasing the phase contrast. Clearly, POM and COPA phases form co-continuous structure and both the surface and cross-section of COPA phase can be observed. A high magnification image (Figure 1d) shows a gap with a width of about 600 nm measured using the image analysis software Smile View (JEOL software, Tokyo, Japan) between the POM and COPA phases, which is caused by the removal of the TPU phase. CB particles are observed along the COPA side of the gap, and there are almost no CB particles at the POM side. The high magnification images of the COPA phase (Figure 1b,c) show that the majority of CB particles are localized on the surface of the COPA phase (Figure 1c); although they are also present inside the COPA phase (Figure 1b), its number is much less than those on the surface.

Figure 1e shows the FESEM image of the cryofractured cross-section of the sample after removal of the COPA phase by m-cresol. The CB network is observed around the etched region. The TEM image shown in Figure 1f clarifies that CB particles are mainly localized at the TPU/COPA interface, as revealed by the existence of the thin TPU layer that does not contain CB particles near the POM phase. A minority of CB particles are present in the COPA phase.

**Figure 2:**
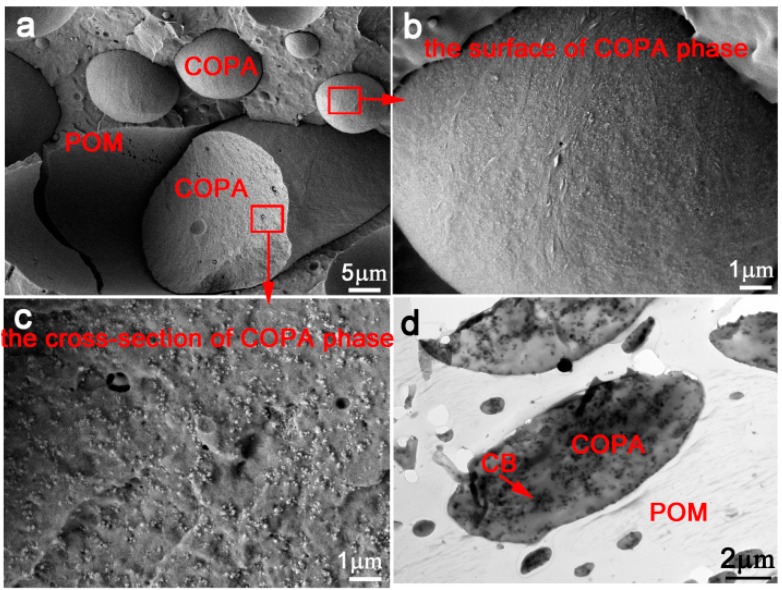
FESEM (**a**–**c**) and TEM (**d**) micrographs of the POM/COPA/CB (70/30/6) composite.

FESEM and TEM images of the sample POM/COPA/CB (70/30/6) without TPU are shown in Figure 2 for comparison. The sea-island structure is observed with POM as the matrix and COPA as the dispersed phase (Figure 2a). CB particles are uniformly distributed in the COPA phase and very little CB particles were observed on the surface of the COPA phase (Figure 1b). This is further confirmed by the TEM micrograph shown in Figure 2d. Therefore, the selective localization of CB particles at the interface of COPA/TPU in the POM/COPA/TPU/CB composite is driven by the minor TPU phase.

The localizations of the minor TPU phase and CB particles were further confirmed by the selective extraction technique [36]. By measuring the weight difference before and after immersion of the POM/COPA/TPU/CB (65/30/5/6) composite in DMF, the proportion of the TPU phase spread at the interface of POM/COPA phases was calculated. The result shows that 93% of the minor TPU component was located at the POM/COPA interface. In particular, the solution after etching was colorless, which indicates that the CB particles were localized at the interface of COPA/TPU, rather than inside the minor TPU phase. This is consistent with the TEM image.

The combined results of electron microscopy and selective extraction technique suggest that the desired hierarchical structure, where minor TPU component spreads at the interface of two major components (POM/COPA) to form tri-continuous structure and CB particles are selectively localized at the interface of COPA and TPU, is formed in POM/COPA/TPU/CB composite prepared by conventional melt mixing technique.

### 3.3. Percolation and Morphology Development as a Function of CB Content

**Figure 3:**
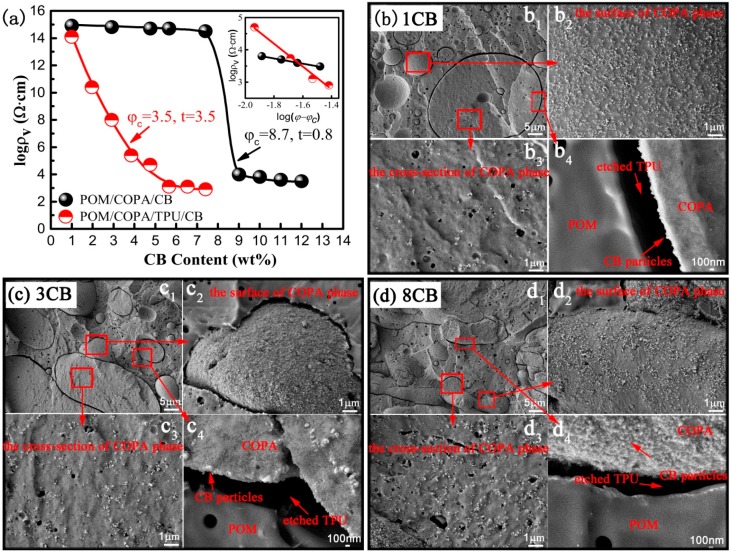
(**a**) Electrical resistivity as a function of CB content for POM/COPA/CB and POM/COPA/TPU/CB composites. The compositions of POM/COPA and POM/COPA/TPU blends were fixed at 70/30 and 65/30/5, respectively. (**b**–**d**) The FESEM images of the POM/COPA/TPU/CB composites with different CB contents: 1 phr (**b**), 3 phr (**c**), and 8 phr (**d**), and the TPU phase was etched by DMF.

It is well-known that the morphology of the conductive networks has an important influence on the electrical properties of the conductive polymer composites, therefore, in this section we display the electrical percolation curves (Figure 3a) of two different morphological composites described in Section 3.1 for comparison. The POM/COPA/TPU/CB composites show a significantly reduced electrical resistivity throughout the whole CB content compared with the POM/COPA/CB composites. According to classical percolation theory, the dependence of electrical resistivity data on the conductive filler content above the percolation threshold ($\varphi_{c}$) can be fitted by a scaling law [37,38]:

(4) $$\sigma =\sigma_{0}{\left(\varphi -\varphi_{c}\right)}^{t}$$

where σ is the electrical conductivity of the composites, σ_0_ is a scaling factor, ϕ is the filler content, and t is the critical exponent. The curves fitting to the scaling law are shown in the inset of Figure 3a. The calculated percolation threshold of POM/COPA/CB composites is 8.7 wt %, while after the incorporation of the minor TPU phase, it decreases to 3.5 wt % with a reduction of 59.8%, revealing that the conductive network composed of CB localized at the interface of COPA/TPU is much more efficient than that composed of CB distributed in the COPA phase.

**Figure 4:**
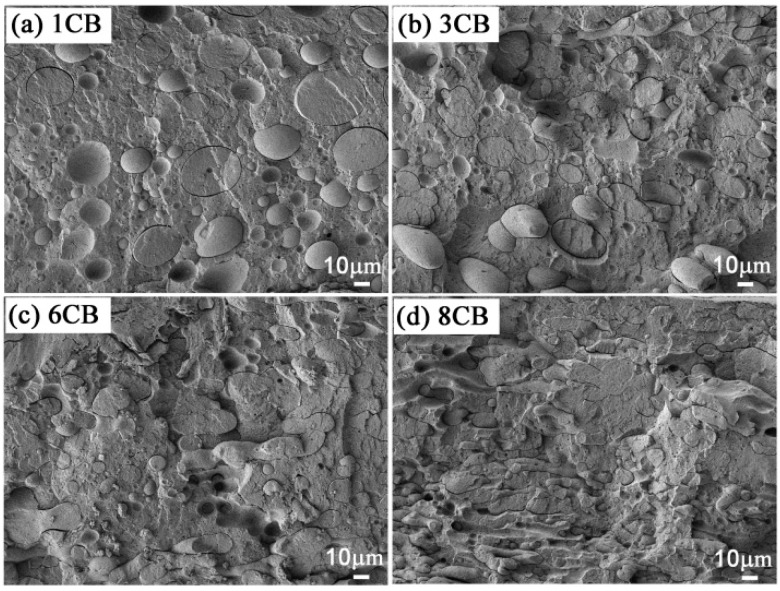
FESEM images of POM/COPA/TPU/CB (65/30/5/*x*) at relatively low magnification with different contents of CB: (**a**) 1 phr, (**b**) 3phr, (**c**) 6 phr, and (**d**) 8 phr. The TPU phase was etched by DMF.

It is commonly known that the selective localization of the filler in the polymer blend may cause morphological changes and affect the final properties of the material [39,40]. Thus, the morphologies of POM/COPA/TPU/CB composites with different CB contents are shown in Figure 3b–d and Figure 4 to show the morphology development as a function of CB content and its relationship with the electrical resistivity. When the CB content is extremely low, there are very few CB particles in the COPA phase (Figure 3b_3_), while the surface of the COPA phase is full of CB particles (Figure 3b_2_), and there are no CB particles at the POM side of the gap left by the removal of TPU (Figure 3b_4_). This further confirms that CB particles are selectively localized at the interface of COPA/TPU. Although the CB content in the COPA phase gradually increases with the increase of CB loading, it is far lower than that on the surface of COPA phase (Figure 3c,d). Figure 4 shows that when CB content is low (1 and 3 phr), the COPA phase mainly exists as islands which are far away from each other. This explains the high electrical resistivity at low CB loading, even if the CB content at the interface of COPA/TPU is rich enough. When CB content increases (6 and 8 phr), the COPA phase becomes continuous. This may be caused by higher intensity forces needed to break the more intense CB network surrounding the COPA phase. The slowdown of the droplet breakup is the mechanism for COPA phase coarsening. This is consistent with the facts that CB self-networking in the dispersed PA6 phase of ABS/PA6 blend induces a co-continuous structure [40], and localization of MWCNTs in the minor PS phase and at the interface of PP/PS blends resulted in the transformation of phase morphology from a sea-island to a co-continuous structure [39]. The electrical resistivities at high CB loadings are low because of the occurrence of double percolation as a result of the formation of tri-continuous phase morphology.

### 3.4. Conductive Networks Formed in Ternary Blends of Different Compositions

**Figure 5:**
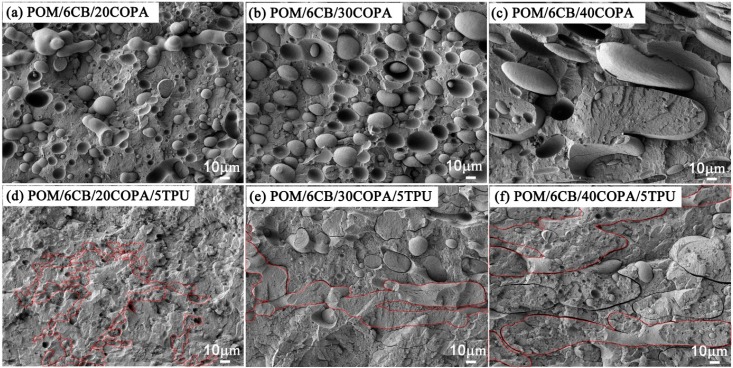
FESEM micrographs of (**a**) POM/6CB/20COPA; (**b**) POM/6CB/30COPA; (**c**) POM/6CB/40COPA; (**d**) POM/6CB/20COPA/5TPU; (**e**) POM/6CB/30COPA/5TPU; and **(f)** POM/6CB/40COPA/5TPU composites. The TPU phase was etched by DMF.

**Figure 6:**
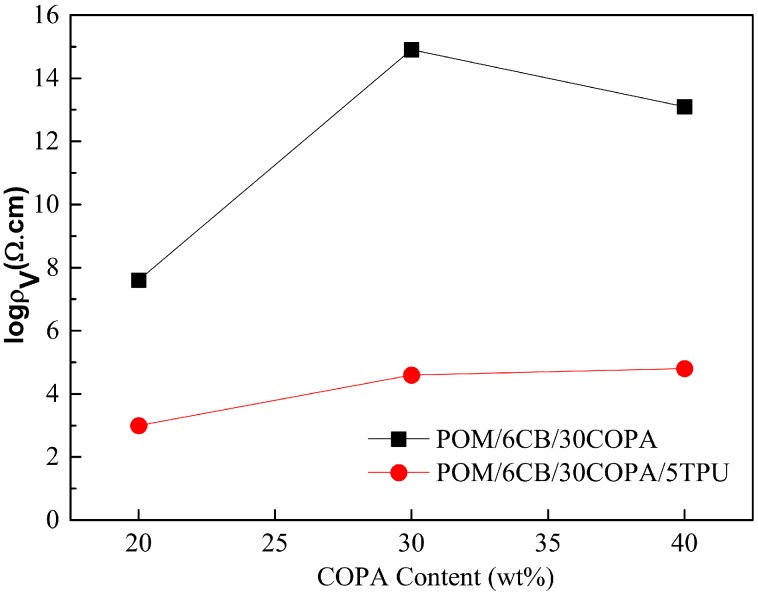
Electrical resistivities of the composites containing different amounts of COPA with and without minor TPU component.

The blend ratio is a key factor affecting the final morphology of the polymer blend formed during mixing. Therefore, in this section the ratio of the POM/COPA was changed in keeping the CB loading constant at 6 wt % and TPU content at 5 wt %, to study the effect of blend composition on the formation of conductive network. The corresponding CB-filled POM/COPA binary blends were also studied under the same conditions for comparison. As shown in Figure 5a–c, all of the composites containing 20, 30 and 40 wt % COPA without minor TPU component show sea-island structure. The electrical resistivities of these samples are relatively high, especially for the samples containing 30 and 40 wt % COPA (Figure 6) due to decreased concentration of CB in the COPA phase. After the addition of minor TPU component (5 wt %), the morphologies of POM/COPA in all the samples shifted towards co-continuous structure (Figure 5d–f) because the CB network at the TPU/COPA interface inhibits the breakup of the COPA phase. As a result, the electrical resistivities of these samples are significantly reduced (Figure 6) due to the formation of the tri-continuous structure and the effect of double percolation.

### 3.5. Mechanism of the Conductive Network Formation

**Figure 7:**
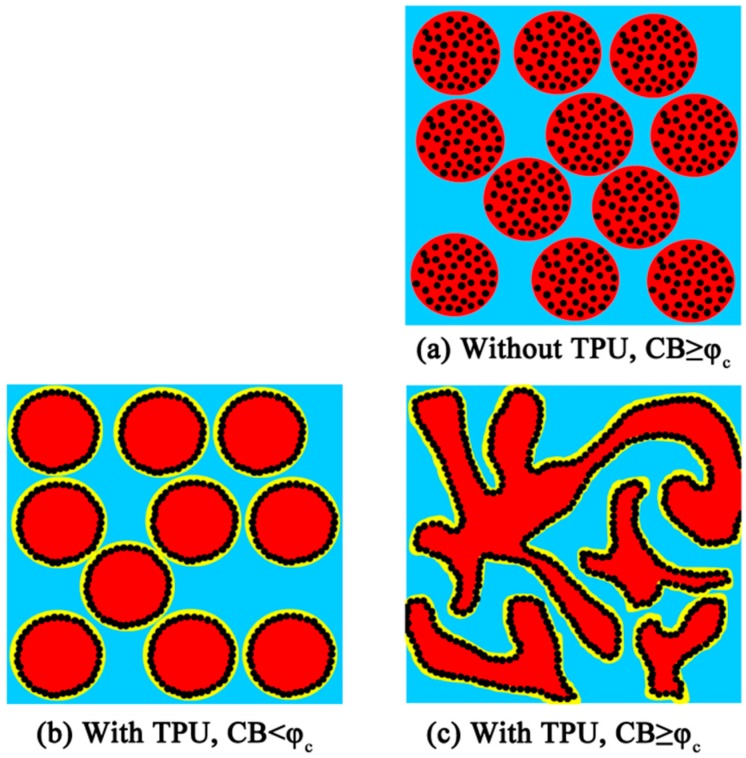
Schematic illustration for the conductive network formation in CB-filled POM/COPA/TPU ternary blends.

In Figure 7, the mechanism of the conductive network formation in POM/COPA/TPU/CB composites is illustrated and contrasted with that of the un-formed conductive network in POM/COPA/CB composites, where ϕ_c_ represents the CB content required to percolate in the POM/COPA/TPU/CB composite. Without TPU, CB forms a conductive network within the dispersed COPA domains when CB content is high enough, however, the blend is not conductive since COPA domains are separated from each other. After introducing a minor TPU component into the composites, the minor TPU phase spreads at the interface of two major components (POM/COPA), and CB particles selectively localized at the interface of COPA/TPU. When CB content is lower than ϕ_c_, COPA/TPU/CB forms core-shell droplets in POM matrix, and the composite is not conductive even the CB concentration is high at the interface of COPA/TPU, since COPA/TPU/CB core-shell droplets are too far apart (Figure 7b). When the content of CB reaches ϕ_c_, the core-shell droplets transform into co-continuous structure and the ternary blend is tri-continuous; CB particles construct a conductive network at the interface of COPA/TPU and the composite is conductive due to the double percolation effect (Figure 7c).

## 4. Conclusions

Incorporating CB to the POM/COPA/TPU ternary blend is a good way to prepare conductive composites with low CB content. Efficient formation of the conductive network is formed by a hierarchical structure composed of a minor TPU phase spreading at the interface of two major continuous phases (POM/COPA) and CB particles selectively localized at the COPA/TPU interface. The percolation threshold of CB particles reduces by 60% compared to that in the corresponding POM/COPA binary blend without minor TPU component, where CB is selectively distributed in the COPA phase.

The CB network at the COPA/TPU interface inhibits the breakup of the COPA phase and induces the formation of tri-continuous phase morphology beyond a critical CB content. The phase inversion of the two major phases from the sea-island to co-continuous structure at the percolation threshold of CB and construction of the CB network at the TPU/COPA interface is the mechanism for the formation of the conductive network.

This work demonstrates that instead of using a copolymer to drive the conductive particles to the interface of binary blends, using an ordinary polymer, such as TPU, can also achieve a favorable hierarchical structure to allow double percolation to occur. This opens up the possibility of constructing efficient conductive networks in a variety of polymer blends.

## Figures and Tables

**Table 1:** Surface tension (γ) and the surface tension components (γ^d^, γ^p^) and (–dγ/dT) for polymers and CB at different temperatures.

Components	γ (20 °C) (mJ/m^2^)	γ (200 °C) (mJ/m^2^)	γ^d^ (200 °C) (mJ/m^2^)	γ^p^ (200 °C) (mJ/m^2^)	−dγ/dT mJ/(m^2^°C)
POM [30]	44.6	30.7	21.0	9.7	0.077
COPA	47.4	32.6	29.1	3.5	0.082
TPU [31]	44.0	30.3	22.5	7.8	0.076
CB [32,33]	98.1	87.3	84.1	3.2	0.060

**Table 2:** Interfacial tensions, spreading coefficients and wetting coefficients at 200 °C.

Component couple	Interfacial tension (mJ/m^2^)	Spreading coefficient (mJ/m^2^)	Wetting coefficient for CB	Localization
POM-COPA	4.2	λ_POM/TPU/COPA_ = 1.5	ω_POM/COPA_ = –3.4	COPA Phase
POM-TPU	0.3	λ_POM/COPA/TPU_ = −6.4	ω_POM/TPU_ = –13.3	TPU Phase
COPA-TPU	2.5	λ_TPU/POM/COPA_ = −2.0	ω_TPU/COPA_ = −4.4	COPA Phase
POM-CB	41.2			
COPA-CB	26.7			
TPU-CB	37.5			
